# Mechanisms of fungal pathogenic DNA-activated STING pathway in biofilms and its implication in dental caries onset

**DOI:** 10.3389/fcimb.2025.1666965

**Published:** 2025-09-01

**Authors:** Yujie Zhou, Huanzhong Ji, Yuzhe Zhang, Yukun Liu, Yang Ning, Ping Li

**Affiliations:** ^1^ Hospital of Stomatology, Guangdong Provincial Key Laboratory of Stomatology, Guanghua School of Stomatology, Sun Yat‐sen University, Guangzhou, China; ^2^ Department of Prosthodontics, School and Hospital of Stomatology, Guangzhou Medical University, Guangzhou, Guangdong, China; ^3^ School and Hospital of Stomatology, Guangdong Engineering Research Center of Oral Restoration and Reconstruction, Guangzhou Medical University, Guangzhou, Guangdong, China

**Keywords:** STING pathway, fungal biofilms, extracellular DNA, dental caries, innate immunity

## Abstract

Dental caries, a prevalent oral disease, has long been attributed primarily to bacteria, but emerging evidence highlights the critical role of fungi in its pathogenesis. Fungal biofilms, predominantly *Candida albicans*, release extracellular DNA (eDNA) and DNA-carrying extracellular vesicles (EVs). Together with bacterial eDNA, these form the biofilm matrix and can activate the host cGAS-STING signaling pathway. This review systematically elaborates on the molecular architecture and biological functions of the cGAS-STING pathway, comparing mechanistic differences in its activation by viral, bacterial, and fungal DNA. It further explores direct and indirect modes of STING pathway activation by fungal eDNA and EV-carried DNA, along with their immunoregulatory roles. Specifically, it discusses the interactive mechanisms between fungal biofilms and STING activation in root caries onset, emphasizing the dual effects of STING-mediated immune responses—enhancing antifungal immunity while potentially exacerbating tissue damage via excessive inflammation. Finally, this review outlines current knowledge gaps and future research directions, aiming to provide novel insights for precision prevention and treatment of dental caries.

## 1 Introduction

Dental caries is a highly prevalent chronic oral infectious disease worldwide, with its high incidence and disabling impact posing a serious threat to human oral health and quality of life (Wen et al., 2022; Yirsaw et al., 2024). For a long time, it has been widely accepted that acid production metabolism and biofilm formation by cariogenic bacteria like *Streptococcus mutans* are the core mechanisms of dental caries. Related antibacterial strategies have mostly focused on bacteria (Bhat et al., 2023; Jiang et al., 2023). However, with the development of microbiome technologies in recent years, growing evidence indicates that fungi represented by *Candida albicans* play an undeniable role in the occurrence and development of dental caries, especially root caries (Ji et al., 2025; Zhang et al., 2025).

These fungi, together with bacteria, form complex mixed biofilms at carious sites. The extracellular DNA (eDNA) they secrete not only cross-links with bacterial eDNA to form the biofilm matrix skeleton—enhancing community stability and stress resistance—but also acts as a key signaling molecule in host immune regulation (Gallucci, 2024; Geng et al., 2024; Han et al., 2025). Among these, the cGAS-STING signaling pathway serves as a core innate immune hub for cytosolic DNA recognition. The molecular mechanisms by which fungal eDNA and vesicle-carried DNA activate this pathway are critical. They link fungal biofilm colonization to host immune response imbalance (Brown Harding et al., 2024; Yang et al., 2024).

This review systematically clarifies the specific mechanisms by which pathogenic DNA in fungal biofilms—including eDNA and vesicle-carried DNA—activates the STING pathway. It analyzes how these mechanisms regulate immune balance in the caries microenvironment and influence dental hard tissue destruction. It also outlines future research directions. This work lays a theoretical foundation for advancing the understanding of caries etiology and facilitating the development of precise preventive and therapeutic strategies.

## 2 Molecular architecture and biological functions of the STING signaling pathway

The cGAS-STING signaling pathway acts as a central hub linking cytosolic DNA recognition to innate immune responses (Decout et al., 2021). It plays pivotal roles in defending against pathogenic invasion, regulating anti-tumor immunity, and maintaining autoimmune homeostasis.Cyclic GMP-AMP synthase (cGAS) is a cytosolic DNA sensor, primarily distributed in the cytoplasm and nucleus. Its N-terminal DNA-binding domain (DBD) specifically recognizes double-stranded DNA (dsDNA), including pathogenic DNA, damaged nuclear DNA, and mitochondrial DNA (Gentili et al., 2019). Upon binding to dsDNA, cGAS undergoes conformational changes to activate its C-terminal catalytic domain. This catalyzes the synthesis of the second messenger 2’,3’-cyclic GMP-AMP (2’,3’-cGAMP) from ATP and GTP.As a unique cyclic dinucleotide (CDN), 2’,3’-cGAMP diffuses from its binding site. It then interacts with the transmembrane domain of STING (stimulator of interferon genes), which is localized on the endoplasmic reticulum (ER) (Yu and Liu, 2021; Liu et al., 2022). This interaction induces STING tetramerization and translocation from the ER to the Golgi apparatus and perinuclear vesicles. During translocation, STING recruits the serine/threonine kinase TBK1 (TANK-binding kinase 1). TBK1 phosphorylates the C-terminal tail (CTT) of STING. Activated STING further mediates TBK1-dependent phosphorylation of transcription factors IRF3 (interferon regulatory factor 3) and NF-κB (nuclear factor κB). This drives their nuclear translocation, inducing the expression of type I interferons (IFN-α/β), proinflammatory cytokines (e.g., IL-6, TNF-α), and antiviral proteins (ISGs). This completes the signal cascade amplification (Kwon and Bakhoum, 2020).

Functionally, the cGAS-STING pathway has extensive biological significance. In antiviral immunity, it recognizes viral DNA released by herpesviruses and poxviruses. This induces type I interferons to inhibit viral replication (Decout et al., 2021; Zhang et al., 2024). It also activates innate immune cells such as dendritic cells and macrophages, promoting antigen presentation and initiating adaptive immune responses (Ou et al., 2021; Zhou et al., 2023). In tumor immunoregulation, the pathway recognizes DNA released by necrotic tumor cells. When activated via STING agonists, it enhances the infiltration and activation of cytotoxic T lymphocytes (CTLs). This induces tumor cell apoptosis or autophagy (Samson and Ablasser, 2022). Currently, STING agonists are in clinical trials for melanoma and lung cancer. Their combination with PD-1 inhibitors or chemotherapeutic drugs significantly improves anti-tumor efficacy (Pan et al., 2023; Wang et al., 2024).

However, aberrant activation of the cGAS-STING pathway is linked to various diseases. In autoimmune disorders, self-DNA (e.g., nucleosomal DNA in systemic lupus erythematosus patients) is misrecognized by cGAS. This leads to sustained STING activation and excessive type I interferon production, triggering autoimmune reactions (Zierhut and Funabiki, 2020; Skopelja-Gardner et al., 2022; Liu and Pu, 2023). Patients with Aicardi-Goutières syndrome exhibit abnormal pathway activation due to mutations in cGAS or STING. This results in severe type I interferonopathy (Crow and Manel, 2015). Additionally, mitochondrial DNA leakage into the cytoplasm (e.g., during ischemia-reperfusion injury or neurodegenerative diseases) activates the pathway (Paul et al., 2021; Guo et al., 2024b). This exacerbates inflammatory responses and tissue damage. The pathway also participates in cellular responses to DNA damage. It regulates cell cycle arrest, apoptosis, pyroptosis, and senescence, thereby influencing tissue repair and organismal aging (Bai and Liu, 2019; Schmitz et al., 2023).

## 3 Mechanistic differences in STING pathway activation by viral and bacterial DNA

Host recognition and clearance of pathogenic microorganisms form a critical defense line of the immune system. Innate immunity acts as the first and fastest barrier against invading microbes. The DNA-activated STING pathway is a key immune mechanism for pathogen recognition. Viruses and bacteria use distinct strategies to deliver DNA into the host cytoplasm, triggering the pathway and inducing immune responses. During viral infection, enveloped viruses (e.g., herpesviruses, poxviruses) release dsDNA into the cytoplasm via membrane fusion or endocytosis. cGAS specifically recognizes the double-stranded structure, length, and conformational features of viral DNA through its N-terminal DBD. Upon binding, it activates the C-terminal catalytic domain to generate 2’,3’-cGAMP.2’,3’-cGAMP then binds to ER-localized STING, inducing its activation and translocation to the Golgi apparatus and perinuclear vesicles. This is followed by TBK1 recruitment, which phosphorylates IRF3 and NF-κB. Ultimately, this induces type I interferons and proinflammatory cytokines (Wu et al., 2022; Patel et al., 2023).Notably, some viruses (e.g., adenoviruses) bypass cGAS. They activate STING via direct binding or interference with nucleic acid metabolism (Lam et al., 2014). Retroviral cDNA intermediates and RNA virus-induced mitochondrial DNA leakage also indirectly activate the pathway. To evade immunity, viruses encode proteins that degrade cGAS or inhibit STING translocation (Lange et al., 2022; Xie and Zhu, 2024).

Bacterial activation of the STING pathway differs mechanistically. Intracellular bacteria (e.g., *Listeria monocytogenes*) secrete hemolysins to disrupt phagosomal membranes. This releases bacterial DNA rich in unmethylated CpG motifs into the cytoplasm. These motifs enhance DNA-cGAS binding affinity, promoting 2’,3’-cGAMP production (Glomski et al., 2002; Fehér, 2019). Extracellular bacteria (e.g., *Escherichia coli*) inject DNA into host cells via lysis or type III secretion systems (Cornelis, 2000). Beyond the canonical cGAS-dependent pathway, some bacterial DNA is recognized by endosomally localized TLR9. This synergizes with the STING pathway to activate NF-κB and IRF3, amplifying proinflammatory responses (Temizoz et al., 2022; Danielson et al., 2024). However, bacteria also regulate the STING pathway. For example, *Mycobacterium tuberculosis* DNA activates STING, but its cell wall components inhibit STING translocation. This attenuates interferon responses to facilitate survival (Marinho et al., 2017; Sun et al., 2020).

In summary, viral and bacterial activation of the STING pathway both start with cGAS recognition of cytosolic DNA. But they differ significantly in DNA sources, cytosolic delivery modes, recognition priorities, downstream effects, and evasion mechanisms. Viruses primarily rely on their genomic DNA or replication intermediates to activate the pathway, inducing type I interferons to inhibit replication (Zevini et al., 2017). Bacteria deliver DNA via phagosomal disruption or secretion systems, synergizing with other pathways to enhance phagocytic killing (Ryan et al., 2023). These mechanisms highlight the complexity of host-pathogen interactions.

A comparative summary of the key mechanisms underlying STING pathway activation by different pathogens is provided in **Table 1**. Among these pathogens, fungi exhibit unique activation modes of the STING pathway, which are discussed in detail below.

**Table 1:** Key mechanisms of STING pathway activation by different pathogens (viruses, bacteria, fungi).

Mechanism Category	Viruses	Bacteria	Fungi (represented by *Candida albicans*)	Reference Sequence
DNA Source	Viral genomic DNA or replication intermediates	Bacterial genomic DNA (released into the cytoplasm by intracellular bacteria, and delivered by extracellular bacteria through secretion systems)	Extracellular DNA (eDNA), genomic DNA/mtDNA fragments carried by extracellular vesicles (EVs)	Cornelis, 2000; Bolognesi and Hayashi, 2011; Lam et al., 2014; Rodrigues et al., 2025
Cytoplasmic Delivery Mode	Envelope fusion with host cell membrane, endocytosis	Intracellular bacteria: disruption of phagosomal membrane; Extracellular bacteria: injection via type III secretion system	eDNA: endocytosis, host cell membrane damage; EVs: membrane fusion or endocytosis	Cornelis, 2000; Glomski et al., 2002; Mayer et al., 2013; Lam et al., 2014; Brown Harding et al., 2024; Rodrigues et al., 2025
Host Recognition Receptor	Primarily dependent on cGAS (some viruses can directly bind to STING)	Primarily dependent on cGAS, with some synergy with endosomal TLR9	Primarily dependent on cGAS (requires recognition of double-stranded structure and unmethylated CpG motifs)	Lam et al., 2014; Fehér, 2019; Jannuzzi et al., 2020; Decout et al., 2021; Temizoz et al., 2022
Activation Auxiliary Factors	Length and conformation of viral DNA (e.g., double-stranded structure)	Unmethylated CpG motifs in bacterial DNA (enhancing binding affinity with cGAS)	Accumulation of high-concentration eDNA, phagosomal rupture (*Cryptococcus neoformans*), release of host mtDNA (*Aspergillus fumigatus*), synergy with CLRs and other PRRs	Wagener et al., 2018; Fehér, 2019; Jang et al., 2022; Kim et al., 2023
Escape Strategies	Encoding proteins to degrade cGAS, inhibiting STING translocation	*Mycobacterium tuberculosis*: inhibiting STING translocation; degrading host recognition receptors	Methylation of CpG motifs (reducing affinity with cGAS), secreting proteases to degrade STING, formation of eDNA-polysaccharide complexes to hinder recognition	Gropp et al., 2009; Lam et al., 2014; Valiante et al., 2015; Sun et al., 2020; Jang et al., 2022; Massey et al., 2023; Zhang et al., 2023
Main Immune Effects	Inducing type I interferons (IFN-α/β) to inhibit viral replication	Inducing proinflammatory cytokines (IL-6, TNF-α) to enhance phagocytic bactericidal activity	Inducing type I interferons and proinflammatory cytokines to enhance antifungal immunity; sustained activation may lead to excessive inflammation and tissue damage	Decout et al., 2021; Salgado et al., 2021; Samson and Ablasser, 2022; Temizoz et al., 2022; Brown Harding et al., 2024; Gallucci, 2024; Sun et al., 2024

## 4 Activation modes and immunoregulation of host STING pathway in fungal infections

During fungal infections, host cells recognize pathogens and initiate immune responses via multiple signaling pathways. Among these, the STING pathway interacts intricately with other immune signaling networks (Chen et al., 2023). As eukaryotic pathogens, fungi exhibit unique and diverse mechanisms for activating the STING pathway, emerging as a research hotspot in recent years.

In direct activation, invasive fungi (e.g., *Candida albicans*) release genomic DNA into the host cytoplasm. This occurs via secreted hydrolases (which disrupt cell membranes) or phagosomal rupture (Naglik et al., 2003; Mayer et al., 2013). Host cGAS recognizes the double-stranded structure, unmethylated CpG motifs, or specific conformational features (dsDNA length >40 bp) of fungal DNA. It then catalyzes 2’,3’-cGAMP synthesis. 2’,3’-cGAMP binds to ER-localized STING, inducing conformational changes and translocation to the Golgi apparatus. This is followed by TBK1 recruitment, which phosphorylates IRF3 and NF-κB. Ultimately, this induces the expression of type I interferons (e.g., IFN-β) and proinflammatory cytokines (e.g., IL-6, TNF-α), thereby enhancing antifungal immunity (Yu and Liu, 2021; Yum et al., 2021; Su et al., 2022). However, as eukaryotic DNA, fungal DNA has higher CpG methylation levels. Theoretically, it has lower cGAS binding affinity than bacterial DNA. Thus, it requires higher concentrations or specific conditions (e.g., repeated infections, phagosomal rupture) for effective activation (Jeon et al., 2015; Sarkies, 2022). For example, capsular polysaccharides of *Cryptococcus neoformans* promote phagosomal rupture. This increases fungal DNA-cGAS interactions to indirectly enhance STING activation (Liu et al., 2023c).

Non-cGAS-dependent STING activation in fungal infections primarily involves indirect activation via induced host mitochondrial DNA (mtDNA) release (Kim et al., 2023). For instance, hyphal invasion by *Aspergillus fumigatus* causes host mitochondrial damage. Released mtDNA is recognized by cGAS, activating STING. This “self-DNA + pathogen components” dual activation mode amplifies inflammatory responses in chronic infections (e.g., candidemia) (Kim et al., 2023; Peng et al., 2023). Additionally, fungal cell wall components (e.g., β-glucan, mannose) cannot directly activate STING. But they trigger signaling via other pattern recognition receptors (PRRs), synergizing with the STING pathway. Dectin-1, a C-type lectin receptor (CLR), recognizes β-glucan and activates NF-κB via the Syk-Card9 pathway. STING-induced IFN-β upregulates Dectin-1 expression, enhancing phagocytic bactericidal capacity. This “STING-IFN-other PRRs” cascade integrates antifungal immune signals (Wagener et al., 2018; Salgado et al., 2021).

The STING pathway is indispensable for antifungal immunity. On one hand, type I interferons induced by its activation enhance natural killer (NK) cell and T cell activation. They also promote macrophage phagocytosis and bactericidal function (Chen et al., 2023). On the other hand, proinflammatory cytokines recruit neutrophils, forming an inflammatory barrier to restrict fungal diffusion (Robertson et al., 2017). In animal studies, STING-deficient mice show increased susceptibility to *Candida albicans* and *Aspergillus fumigatus* infections. They exhibit elevated fungal burden and exacerbated tissue damage, confirming STING’s critical role in host antifungal immunity (Chen et al., 2023). Moreover, the STING pathway interacts with autophagy. STING activation induces autophagy-related genes (e.g., ATG5, ATG7), promoting phagosome-lysosome fusion to accelerate fungal degradation (Liu et al., 2019; Schmid et al., 2024). Autophagy also clears intracellular fungal DNA, avoiding excessive STING activation-induced immunopathological damage. This balances antifungal efficacy and tolerance (Maluquer De Motes, 2022).

Fungi have evolved multiple strategies to evade the STING pathway. At the DNA level, fungi such as *Blastomyces dermatitidis* methylate CpG motifs in their DNA, reducing cGAS binding efficiency (Yu and Liu, 2021). *Cryptococcus neoformans* capsular polysaccharides encapsulate DNA, blocking cGAS recognition (Jang et al., 2022). At the signaling level, *Candida albicans* secretes aspartic proteases (e.g., Sap2) that degrade host STING, inhibiting ER-to-Golgi translocation (Gropp et al., 2009). Mannoproteins in *Aspergillus fumigatus* cell walls competitively bind 2’,3’-cGAMP with STING, blocking downstream signaling (Valiante et al., 2015). Additionally, fungi such as *Talaromyces marneffei* inhibit host mtDNA release, reducing cGAS substrates to attenuate STING pathway responses (Zhang et al., 2023).

## 5 Fungal extracellular DNA: a potential bridge from biofilm matrix to STING pathway activation

Fungal extracellular DNA (eDNA) is a key component of fungal biofilms. It has emerged as a research focus due to its interactions with the host immune system and potential to activate the STING pathway during infections. eDNA refers to DNA actively secreted by fungi or released into the extracellular environment upon cell lysis. It is widely present in biofilm matrices of pathogenic fungi such as *Candida albicans* and *Aspergillus fumigatus* (Rajendran et al., 2013; Juszczak et al., 2024). It is generated via two main pathways: active secretion (dependent on specific fungal secretion systems) and passive release (from cell wall/membrane rupture induced by programmed cell death, mechanical damage, or host immune attacks) (Bolognesi and Hayashi, 2011). Fungal eDNA has double-stranded structures and contains unmethylated CpG motifs, providing a structural basis for recognition by host pattern recognition receptors (Panchin et al., 2016).

Fungal eDNA has multiple biological functions. In biofilm construction and protection, eDNA forms a three-dimensional network via physical cross-linking. It connects hyphae, yeast cells, and extracellular polysaccharides to enhance mechanical stability. It also defends against antifungal drug penetration and host immune clearance. For example, *Candida albicans* eDNA chelates echinocandins, reducing their inhibition of cell wall β-glucan synthase (Rajendran et al., 2013; Sharma and Rajpurohit, 2024). In regulating fungal physiology and virulence, eDNA acts as a signaling molecule to modulate morphological transitions and virulence factor expression. Its specific sequences bind fungal transcription factors, promoting invasion-related gene expression (Campoccia et al., 2021). Additionally, eDNA mediates intercellular communication. It transfers genetic information via horizontal gene transfer, accelerating the spread of drug resistance or virulence genes among fungal populations (Gonçalves and Gonçalves, 2022).

In host interactions, fungal eDNA both activates innate immunity and participates in immune evasion and pathological damage. It is recognized by host surface or intracellular pattern recognition receptors. Endosomally localized TLR9 recognizes unmethylated CpG motifs in eDNA, activating NF-κB to induce proinflammatory cytokines. cGAS, as a cytosolic DNA sensor, also potentially recognizes eDNA (Jannuzzi et al., 2020). Conversely, high eDNA concentrations inhibit immune functions via multiple mechanisms. These include chelating antimicrobial peptides and immune cell surface receptors to impair phagocytosis, or promoting macrophage polarization toward an anti-inflammatory phenotype to reduce antifungal efficiency (Massey et al., 2023). In chronic fungal infections, sustained eDNA stimulation may induce cytokine storms and tissue damage (Conti et al., 2018).

The potential mechanisms of fungal eDNA-activated STING pathway have attracted significant attention, involving direct and indirect activation. In direct activation, eDNA may enter the cytoplasm via endocytosis or host membrane damage. cGAS recognizes its double-stranded structure and unmethylated CpG motifs, catalyzing 2’,3’-cGAMP production. This potentially activates STING, recruits TBK1, phosphorylates IRF3 and NF-κB, and induces type I interferons and proinflammatory cytokines (Liu et al., 2023a; Li et al., 2025). In indirect activation, eDNA may induce host cell damage, promoting mitochondrial DNA (mtDNA) release into the cytoplasm. Or it may synergize with TLR9 and other pattern recognition receptors to enhance STING signaling (Lou and Pickering, 2018; Liu et al., 2023b). Activation efficiency may be influenced by multiple factors. eDNA length, methylation status, and CpG density may affect cGAS binding affinity (Ben Maamar et al., 2023; Dong et al., 2025). eDNA-polysaccharide/protein complexes in biofilms may hinder recognition, while local high concentrations may increase activation probability (LuTheryn et al., 2023). Differences in cGAS-STING pathway sensitivity among host cell types may also impact activation (Li and Bakhoum, 2022).

As a core biofilm component, fungal eDNA’s potential to activate the STING pathway reveals novel interaction modes between fungal infections and host immunity. Targeting the eDNA-STING axis may offer new antifungal strategies, such as developing eDNA-degrading nucleases or STING agonists to enhance immunity. However, eDNA-mediated excessive STING activation may contribute to chronic inflammation and autoimmune diseases, necessitating further research into its regulatory mechanisms in pathological states.

## 6 Mechanisms and immunoregulatory roles of fungal extracellular vesicle–carried DNA in STING pathway activation

Fungal extracellular vesicles (EVs) are membrane-bound vesicles (30–1000 nm in diameter) actively secreted by fungal cells. Their carried DNA plays a key role in host-pathogen interactions (Rodrigues et al., 2025). EV DNA primarily includes genomic DNA fragments, mitochondrial DNA, and cDNA derived from non-coding RNA. Most are in double-stranded or circular forms; some contain unmethylated CpG motifs, endowing potential for recognition by host pattern recognition receptors (Guo et al., 2024a; Ghanam et al., 2025). In pathogenic fungi such as *Candida albicans* and *Aspergillus fumigatus*, DNA constitutes 10%-15% of total EV content. EV secretion and DNA loading efficiency increase significantly during biofilm formation or environmental stress (Ullah et al., 2023).

Functionally, fungal EV DNA has diverse biological significance. In intercellular communication and genetic information transfer, EVs act as “molecular carriers” to transport DNA to recipient fungal cells. This enables horizontal gene transfer, accelerating the spread of drug resistance or virulence genes (Marcilla and Sánchez-López, 2022; Werner Lass et al., 2024). In regulating fungal physiology and virulence, EV DNA modulates gene expression in recipient fungi. For example, Aspergillus fumigatus EV DNA regulates morphology-related genes to promote hyphal growth and enhance invasiveness (Rizzo et al., 2023). Additionally, EV DNA has dual roles in immune regulation. It acts as a pathogen-associated molecular pattern (PAMP) to activate host immunity. It also mediates immune suppression via associated immunomodulatory components to facilitate fungal evasion (Montanari Borges et al., 2024).

In host interactions, fungal EV DNA has complex immunoregulatory properties. EVs enter host cells via endocytosis or membrane fusion, releasing DNA that is recognized by intracellular pattern recognition receptors. Endosomally localized TLR9 recognizes unmethylated CpG motifs in EV DNA, activating NF-κB to induce proinflammatory cytokines (Higuchi et al., 2024). Conversely, some fungal EVs evade immunity. For example, *Cryptococcus neoformans* EV DNA binds immunosuppressive miRNAs to downregulate host cell surface MHC-II expression, impairing antigen presentation (Sk Md et al., 2020). Sustained EV DNA stimulation also induces cytokine imbalance, leading to tissue damage (Fan et al., 2025).

The direct activation pathway of STING by fungal EV-carried DNA likely involves EV entry into host cells via membrane fusion or endocytosis, followed by DNA release. Cytosolic cGAS recognizes this DNA, potentially initiating the 2’,3’-cGAMP-STING signaling cascade to induce antiviral immune responses (Brown Harding et al., 2024; Kwaku et al., 2025). Indirect activation may involve EV DNA-induced host mitochondrial damage, releasing mtDNA to activate cGAS. Or it may synergize with EV-carried components (e.g., β-glucan) via TLR pathways to enhance STING signaling (Tao et al., 2024). This process is regulated by multiple factors. EV DNA fragment length, methylation status, and CpG motif density may affect cGAS recognition efficiency. EV source cell types, membrane components, and loaded immunomodulatory molecules may alter DNA delivery efficiency. Host cell type differences influence STING pathway responsiveness, and EV membrane proteins may regulate STING subcellular localization and activation kinetics.

## 7 Interactive mechanisms between fungal biofilms and STING pathway activation in root caries onset

In the pathogenesis of dental caries, particularly root caries, interactions between fungal biofilms and the STING signaling pathway may form a potential pathogenic hub. At carious sites, *Candida albicans* dominates fungal biofilms and releases extracellular DNA (eDNA) and DNA-carrying extracellular vesicles (EVs). These are recognized by host intracellular cGAS, activating the STING pathway (Tian et al., 2022; Lattar et al., 2024; Han et al., 2025). *Candida albicans* eDNA, together with eDNA from cariogenic bacteria (e.g., *Streptococcus mutans*, *Lactobacillus*), forms a mixed biofilm matrix via physical cross-linking. This creates a dense three-dimensional network that enhances stability and acid tolerance, synergistically producing acids to exacerbate dental hard tissue demineralization (Sampaio et al., 2019; Evans et al., 2025; Han et al., 2025).

**Figure 1:**
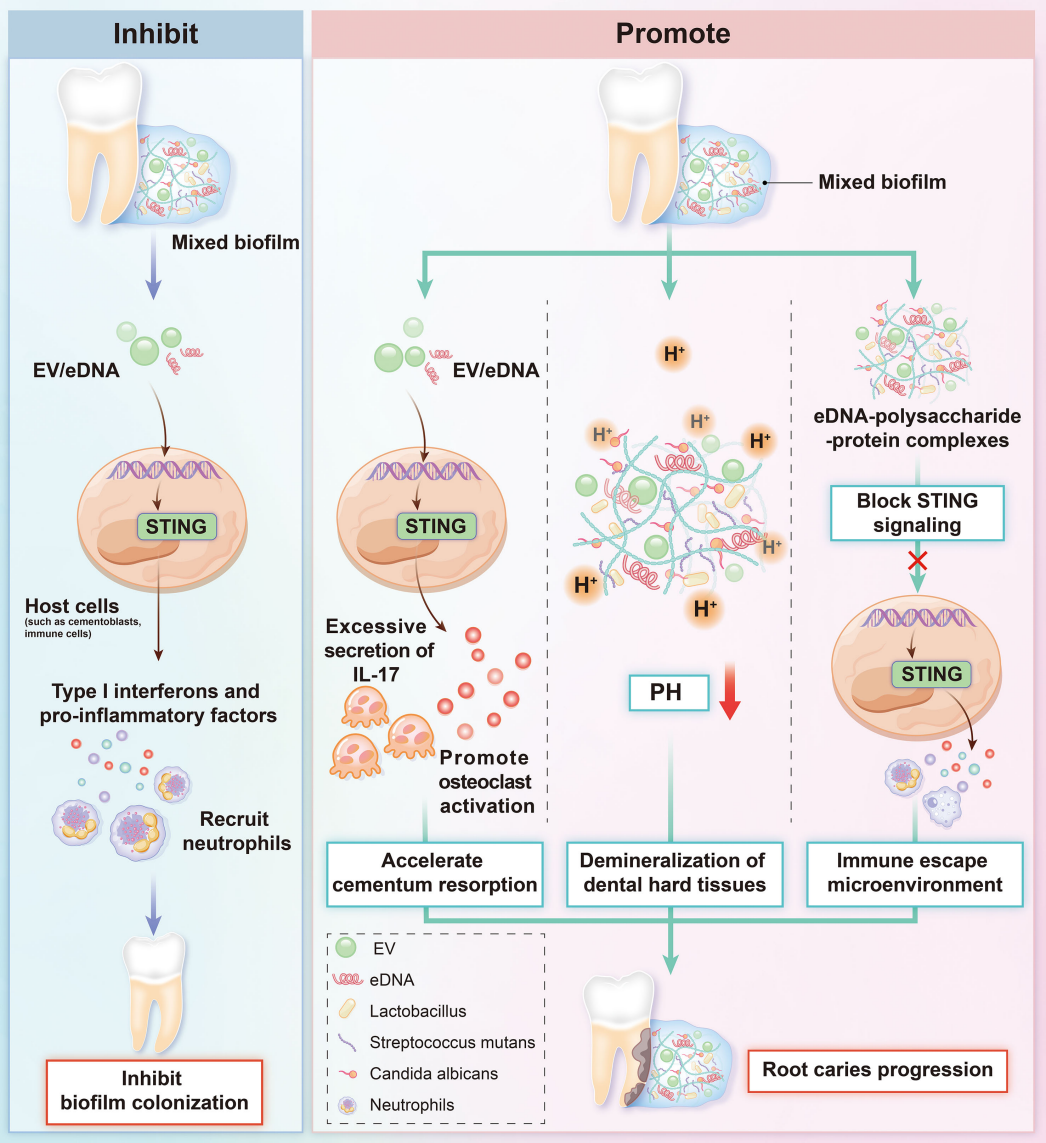
Diagram of the interaction between fungal biofilms and the STING pathway in root caries.

Immunologically, eDNA and EV DNA from mixed biofilms may enter the cytoplasm via endocytosis or membrane damage. This activates the STING pathway, inducing type I interferons and proinflammatory cytokines (Gallucci, 2024). This immune response has dual effects. On one hand, STING activation enhances antifungal immunity to inhibit biofilm colonization. On the other hand, sustained activation may induce excessive secretion of cytokines such as IL-17. This promotes osteoclast activation, accelerates cementum resorption, and drives root caries progression (Mao et al., 2024; Sun et al., 2024). Additionally, eDNA-polysaccharide/protein complexes in the biofilm matrix may block STING signaling, forming an immune-evasive microenvironment (Buzzo et al., 2021). Fungal and cariogenic bacterial metabolic synergies (e.g., carbon source sharing, mutual provision of growth factors) may further amplify inflammatory damage via the STING pathway (Montelongo-Jauregui et al., 2019; Kim et al., 2020). The dynamic interplay between fungal biofilms and the STING pathway offers novel insights into the mechanisms of immune dysregulation and tissue destruction in dental caries (**Figure 1**).

## 8 Conclusion and perspective

Fungal DNA released via multiple pathways—including extracellular DNA (eDNA) and extracellular vesicle (EV)-carried DNA—may activate the cGAS-STING signaling pathway to induce host immune responses. This mechanism may play a unique role in dental caries. eDNA and EV DNA from carious fungal biofilms (dominated by *Candida albicans*) may trigger STING activation. This occurs via direct cGAS stimulation or indirect induction of host mitochondrial DNA release. However, activation efficiency may be influenced by the cariogenic microenvironment (e.g., acidic pH, bacterial metabolites). Additionally, synergies between fungal biofilms and cariogenic bacteria (e.g., enhanced acid production) may amplify inflammatory damage via the STING pathway. Meanwhile, eDNA-polysaccharide complexes in the biofilm matrix may block STING signaling, forming an immune-evasive microenvironment. This complex interplay likely has unique significance in immune dysregulation and dental hard tissue destruction in caries.

Current research has not clarified the precise mechanisms of fungal DNA-activated STING pathway in the caries-specific microenvironment. For example, it remains unclear whether acidic conditions affect cGAS recognition efficiency of fungal DNA, or how the fungal-to-bacterial eDNA ratio in biofilms influences STING activation intensity. Future studies should focus on three areas: (1) developing nucleases targeting eDNA or EV inhibitors in cariogenic fungal biofilms to block excessive STING activation; (2) exploring STING pathway modulators in caries to balance antifungal immunity and inflammatory responses; (3) integrating oral microbiome research to dissect dynamic regulation of the fungal DNA-STING pathway during caries progression, providing a theoretical basis for precision caries prevention and treatment.
